# Hemangiopericytoma of the neck

**DOI:** 10.1186/1746-160X-6-23

**Published:** 2010-09-24

**Authors:** Paraskevi Tsirevelou, Paschalis Chlopsidis, Ifigenia Zourou, Dimitrios Valagiannis, Charalampos Skoulakis

**Affiliations:** 1 ENT Department, "Achillopouleion" General Hospital of Volos, Polymeri 134, 38222 Volos, Greece; 2 Pathology Department, "Achillopouleion" General Hospital of Volos, Polymeri 134, 38222 Volos, Greece

## Abstract

Hemangiopericytoma (HPC) is an exceedingly rare tumor of uncertain malignant potential. Approximately 300 cases of HPC have been reported since Stout and Murray described HPCs as "vascular tumors arising from Zimmerman's pericytes" in 1942. After further characterization, the WHO reclassified HPC as a fibroblastic/myofibroblastic tumor. Long term follow up is mandatory because the histologic criteria for prediction of biologic behavior are imprecise. There are reports of recurrence and metastasis many years after radical resection. The head and neck incidence is less than 20%, mostly in adults.

We report herein a case of HPC resected from the neck of a 74-year-old woman, who presented in our department with a painless right-sided neck mass. The mass was well circumscribed, mobile and soft during the palpation. The skin over the tumor was intact and normal. Clinical diagnosis at this time was lipoma. A neck computer tomography scan showed a large submucosal mass in the neck, which extended in the muscular sites. The tumor was completely removed by wide surgical resection. During surgery we found a highly vascularised tumor. The histopathologic examination revealed a cellular, highly vascularized tumor. The diagnosis was that of solitary fibrous tumor, cellular variant, with haemangiopericytoma-like features. The patient had normal postoperative course of healing and 24 months later she remains asymptomatic, without signs of recurrence or metastases.

## Introduction

Solitary fibrous tumor was first described by Wagner in 1870 [1]. It develops from the cells lining capillaries, pericytes which are small, oval or spindle-shaped cells lining capillaries [2]. They were first described in 1923 by Zimmermann, as specialized cells normally present around amphibian and vertebrate capillaries; they were thought to be modified smooth-muscle cells [3].

## Discussion

### Epidemiology

In 1942 Stout and Murray described nine tumors which were composed of capillary blood vessels with one or more layers of rounded cells arranged about them which cannot be called glomus tumors and suggested hemangiopericytoma (HPC) as properly descriptive name [4]. Two of the described tumors originated in head and neck sites (infraorbital and auricular). In 1949, Stout expanded on his previous work by better delineating the histological details of 25 cases of hemangiopericytoma submitted to him from medical centers around the country. Two of these originated in the head and neck; the first reported case was of nasal HPC, another was in the tongue base [5,6]. Since then, only approximately 300 cases of HPCs have been mentioned in the literature [7].

Over the years, it appeared that this growth pattern was a non-specific one, shared by numerous, unrelated benign and malignant lesions, and that HPC was better considered as a diagnosis of exclusion. Three categories of lesion may now be individualized within the heterogeneous group of HPC like neoplasms. The first category corresponds to those non-HPC neoplasms that occasionally display HPC-like features (e.g. synovial sarcoma). Lesions belonging to the second category show clear evidence of myoid/pericytic differentiation and correspond to true HPCs. They generally show a benign clinical course, and include glomangiopericytoma/myopericytoma, infantile myofibromatosis (previously called infantile HPC), and a subset of sinonasal HPCs. The third category is the solitary fibrous tumor (SFT) lesional group, which includes fibrous-to-cellular SFTs, and related lesions such as giant cell angiofibromas and lipomatous HPCs. In practice, any HPC-like lesion can be allocated to one of these categories, leaving the ill-defined 'haemangiopericytoma' category empty [3].

The behavior of cellular SFT varies both on its clinical presentation and on histological examination. Thereby we may have a tumor with aggressive clinical presentation, with metastases and increased mitotic activity on the histopathological examination or we may have a tumor with a relatively benign behavior, which increases only locally, without giving metastases [8].

Cellular SFT is uncommon mesenchymal tumor, accounting for 1% of all blood vessel-related neoplasms and less than 3-5% of all soft tissue sarcomas [9].

Such a tumor can occur in any site throughout the human body, since there are everywhere capillaries and they have pericytes. Enzinger and Smith [10] evaluated 106 solitary fibrous tumors and concluded that the commonest site is lower extremity (35%) followed by pelvis or retroperitoneum (25%), trunk (14%) and upper extremity (10%). It has also been described occurring in the brain and spine, oesophagus, breast and lung. Comparing the few clinical observations, that exist, the frequency of occurrence in the head and neck is estimated between 16% and 33% of all cellular SFTs occurring in various localizations [11]. In the head and neck region it has been described in the orbit, nasal cavity, oral cavity, jaw, parotid gland, parapharyngeal space, masticator space, jugular foramen, etc. [12]. Cellular SFT may occur in all age groups, predominantly in the 6^th ^and 7^th ^decades, with no sex predilection. The etiology is unknown, although the presence of cellular SFT has been linked to trauma, prolonged steroid use and hormonal imbalance [13].

### Clinical features

Clinically, the cellular SFT usually presents as a painless enlarging mass [2], symptoms being mostly due to pressure on adjacent structures [12]. Various paraneoplastic syndromes have been described in association with cellular SFT, including hypoglycemia, hypophosphatemic osteomalacia and hypertrophic pulmonary osteoarthropathy [14].

### Histopathological features

Cellular forms of SFT resemble what had been called HPC prior to 1990. Usually they have a monotonous appearance, even, moderate to high cellularity, little intervening fibrosis, numerous thin-walled 'stag horn' branching vessels, and round-to-oval monomorphic tumor cell nuclei [15,16]. Immunohistochemically, tend to be less frequently positive for CD34 than fibrous SFT; when positive, the staining is usually less strong than in fibrous SFT and often focal [3]. Criteria of malignancy for SFT include large tumor size (> 50 mm), disseminated disease at presentation, infiltrative margins, high cellularity, nuclear pleomorphism, areas of tumor necrosis and an increased mitotic index (> 4 mitoses per 10 high-power fields (HPF) [17].

### Diferential diagnosis

Diagnosis of highly vascularized tumors in the head and neck is challenging, especially because of the difficulty in differentiating cellular SFTs from other tumors that have prominent vascularization: schwannoma, myofibroblastoma, metastasis from spindle-cell carcinoma, low-grade fibromyxoid sarcoma (especially if myxoid foci are prominent), synovial sarcoma, and malignant peripheral nerve sheath tumor [3]. Angiographic features may help in differentiating cellular SFTs from other hypervascular lesions. Tomography, radiography and angiography are not specific and magnetic resonance imaging reveals a solid mass with isodense contrast in T1 [7].

### Treatment and prognosis

Survival is correlated with the grade, size, and margin status [17]. Previous reports have examined the effect of grade on the prognosis of patients with cellular forms of SFTs occurring at different sites throughout the body. Enzinger and Smith [10] had analyzed 106 cases of cellular SFTs. In one of their reports, 16% of patients had lesions in the head and neck region, and overall survival was 70%. The authors defined a lesion as high grade if it demonstrated more than four mitotic figures per 10 high-power fields, or displayed increased cellularity or necrosis. In tumors with more than four mitotic figures per 10 high-power fields, 10-year overall survival was 29%. In contrast, patients whose tumors demonstrated four or fewer mitotic figures per high-power field had a 10-year overall survival of 77%. Similarly, worse survival was demonstrated for patients with tumors showing evidence of necrosis and tumors greater than 6.5 cm in diameter [10]. Other investigators, however, have not been able to demonstrate this relationship between mitotic activity and survival [18]. The rate of metastases from cellular SFT is low, and most patients do not develop local recurrences. However, in an analysis of 45 cases of cellular SFT of the head and neck reported in the literature, 40% were locally recurrent and 10% showed distant metastases [19]. Metastases are known to occur to the lung, bone, liver, regional lymph nodes and pancreas [20]. Recurrences can often be long delayed. In Enzinger's report, 7 of 16 patients had recurrence after a disease-free interval of 3 years. The recent experience reported from the Memorial Sloan-Kettering Cancer Center found a 93% and 80% 2 and 5-year survival rate, respectively, for classic cellular SFT, but made no mention if histologically malignant tumors were included [21]. The above underscore the need for close long-term follow-up in all patients with cellular SFT-even those with histological low-grade tumors-but paying particular attention to those lesions that have recurred and/or are high-grade lesions on pathological review. Follow-up examination of recurrent lesions should include a chest radiograph.

The treatment of choice for cellular SFT in any location is wide surgical resection, if it is possible. The usefulness of adjuvant radiation therapy has not been fully supported in the literature, although more recent studies suggest that radiation therapy can be used in some situations [5]. In particular, postoperative radiation therapy has been recommended in cases of incomplete surgical removal. The role of chemotherapy in the treatment of cellular SFT has not been clearly determined. Another study from the Memorial Sloan-Kettering Cancer Center found cellular SFT to be responsive to chemotherapy [22]. In particular, adriamycin used alone or in combination was most effective in producing complete and partial remissions in 50% of their cases. Chemotherapy can be useful for preoperative tumor reduction as a postoperative adjunct for tumor metastases and for palliation of locally nonoperative lesions [23]. Perioperative embolization has been suggested as an adjuvant for decreasing tumor vascularity and size [24]. A study by Craven [24] encouraged the use of routine angiography and perioperative embolization to reduce intraoperative hemorrhage. They point to earlier reports where significant hemorrhage and even exanguination occurred.

## Case report

**Figure 1:**
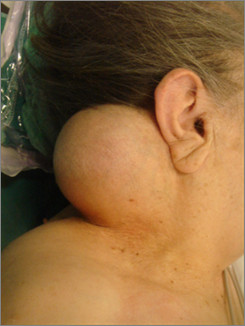
**The patient before surgery**. A 74-year-old female patient presented with a large right-sided neck mass. The skin over the tumor was intact and normal.

**Figure 2:**
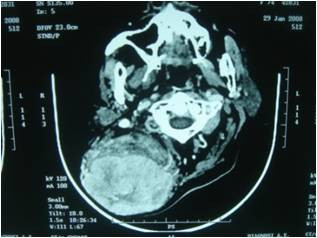
**Computer Tomography scan**. A computer tomography scan showed a large submucosal mass in the neck, 8-9 cm in greatest diameter, extending to the muscular sites.

**Figure 3:**
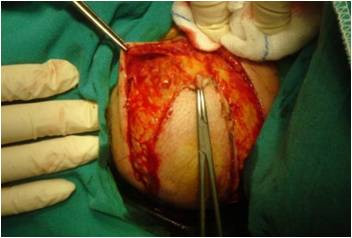
**Surgical resection**. The spindle-shaped incision during surgical resection of the tumor.

A 74-year-old female patient presented in our department with a large right-sided neck mass. The mass was painless, well circumscribed, relatively mobile and soft during the palpation. The skin over the tumor was intact and normal [Fig. 1]. As the patient herself mentioned, the tumor occurred 10 years ago and was increasing gradually. In our case, matches the age and the clinical presentation, as they are described in the literature, but, from our patient's background, there was not anything relevant with the etiology of the tumor's growth, namely, it was not mentioned trauma in the tumor's region nor prolonged steroid use or hormonal imbalance. The reason she came for removal was the aesthetical appearance. Clinical diagnosis at this time was lipoma. A computer tomography scan showed a large submucosal mass in the neck, 8-9 cm in greatest diameter, extending to the muscular sites [Fig. 2]. During surgery under general anaesthesia, it was found that there was not a lipoma, but a sarcomatous tumor with a high vascularisation. The intervention of removal was very earnest, with a big hemorrhage (it was required a blood transfusion of 4 units) [Fig. 3].

Gross examination of the surgical specimen showed a well circumscribed mass with a greatest diameter of 9 cm. Cut surface was yellowish and spongy in appearance.

**Figure 4:**
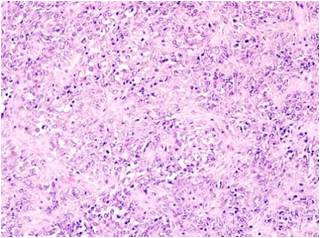
**Histopathological examination**. Histopathological examination of the surgical specimen revealed the presence of solitary fibrous tumor, cellular variant, with haemangiopericytoma-like features (Haematoxilin and Eosin, magnification × 100).

**Figure 5:**
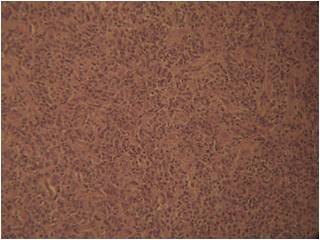
**Histopathological examination**. Monomorphic tumor cells arranged around thin-walled vessels (Hematoxilin and Eosin, magnification × 200).

**Figure 6:**
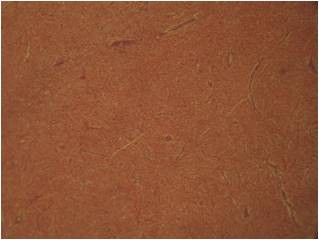
**Histopathological examination**. Masson trichrone stain × 100 - scanty fibrosis.

Microscopy revealed a cellular, highly vascularized neoplasm. The neoplastic cells were closely packed, round or spindle shaped, with scanty cytoplasm and vesicular nuclei, showing little or no pleomorphism. Mitoses rarely exceeded 3 per 10 high-power fields. The vessels were variably ectatic, mostly thin walled and branching. There were also little intervening fibrosis and hemorrhage, as well as some mononuclear inflammatory cells and foamy macrophages [Fig. 4, 5, 6].

**Figure 7:**
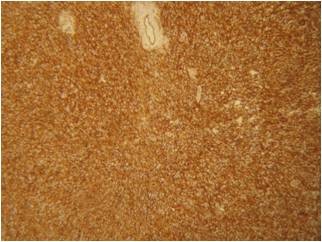
**Histopathological examination**. CD34 immunohistochemical stain: strong positinity of tumor cells.

On immunohistochemical grounds the tumor cells were positive for CD99, CD34 and vimentin and negative for smooth muscle actin, desmin, S-100 and CD31. Ki-67 was < 2% [Fig. 7].

The diagnosis was that of solitary fibrous tumor, cellular variant, with haemangiopericytoma-like features.

**Figure 8:**
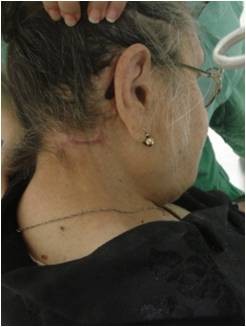
**After surgery**. The patient after surgery.

Our case confirms that the therapeutical standard of cellular SFT is the radical resection and that there is a severe difficulty in the intervention, because of the tumor's high vascularisation and tendency to bleed. The tumor was completely removed by wide surgical resection and our patient had a normal postoperative course without signs of recurrence or metastases, two years later [Fig. 8].

## Conclusions

Cellular variant of SFT is a very rare slow-growing vascular tumor with a variable malignant potential and the biological behavior is difficult to predict. Recommended treatment is wide surgical resection. Long-term follow-up is necessary in patients even after radical resection because recurrence or metastases may be delayed by many years. Adjuvant radiotherapy and chemotherapy can cause tumor regression and are not suggested as primary treatment.

## Competing interests

The authors declare that they have no competing interests.

## Authors' contributions

CS conceived of the study, and participated in its design and coordination and helped to draft the manuscript. PT carried out the drafting of the manuscript and contributed in acquisition of data. PC has made substantial contributions to collection, acquisition and interpretation of data. IZ performed the histopathological examination. DV had the general supervision and have given final approval of the version to be published.

All authors read and approved the final manuscript.

## Consent

Written informed consent was obtained from the patient for publication of this case report and accompanying images. A copy of the written consent is available for review by the Editor-in-Chief of this journal.
